# Cell Death in the Developing Brain after Hypoxia-Ischemia

**DOI:** 10.3389/fncel.2017.00248

**Published:** 2017-08-23

**Authors:** Claire Thornton, Bryan Leaw, Carina Mallard, Syam Nair, Masako Jinnai, Henrik Hagberg

**Affiliations:** ^1^Division of Imaging Sciences and Biomedical Engineering, Centre for the Developing Brain, King's College London, King's Health Partners, St. Thomas' Hospital London, United Kingdom; ^2^The Ritchie Centre, Hudson Institute of Medical Research Clayton, VIC, Australia; ^3^Department of Physiology, Perinatal Center, Institute of Physiology and Neuroscience, Sahlgrenska Academy, University of Gothenburg Gothenburg, Sweden; ^4^Department of Clinical Sciences and Physiology and Neuroscience, Perinatal Center, Sahlgrenska Academy, Gothenburg University Gothenburg, Sweden

**Keywords:** perinatal brain injury, hypoxia-ischemia, mitochondria, apoptosis, necroptosis, necrosis

## Abstract

Perinatal insults such as hypoxia–ischemia induces secondary brain injury. In order to develop the next generation of neuroprotective therapies, we urgently need to understand the underlying molecular mechanisms leading to cell death. The cell death mechanisms have been shown to be quite different in the developing brain compared to that in the adult. The aim of this review is update on what cell death mechanisms that are operating particularly in the setting of the developing CNS. In response to mild stress stimuli a number of compensatory mechanisms will be activated, most often leading to cell survival. Moderate-to-severe insults trigger regulated cell death. Depending on several factors such as the metabolic situation, cell type, nature of the stress stimulus, and which intracellular organelle(s) are affected, the cell undergoes apoptosis (caspase activation) triggered by BAX dependent mitochondrial permeabilzation, necroptosis (mixed lineage kinase domain-like activation), necrosis (via opening of the mitochondrial permeability transition pore), autophagic cell death (autophagy/Na^+^, K^+^-ATPase), or parthanatos (poly(ADP-ribose) polymerase 1, apoptosis-inducing factor). Severe insults cause accidental cell death that cannot be modulated genetically or by pharmacologic means. However, accidental cell death leads to the release of factors (damage-associated molecular patterns) that initiate systemic effects, as well as inflammation and (regulated) secondary brain injury in neighboring tissue. Furthermore, if one mode of cell death is inhibited, another route may step in at least in a scenario when upstream damaging factors predominate over protective responses. The provision of alternative routes through which the cell undergoes death has to be taken into account in the hunt for novel brain protective strategies.

## Introduction

**Figure 1:**
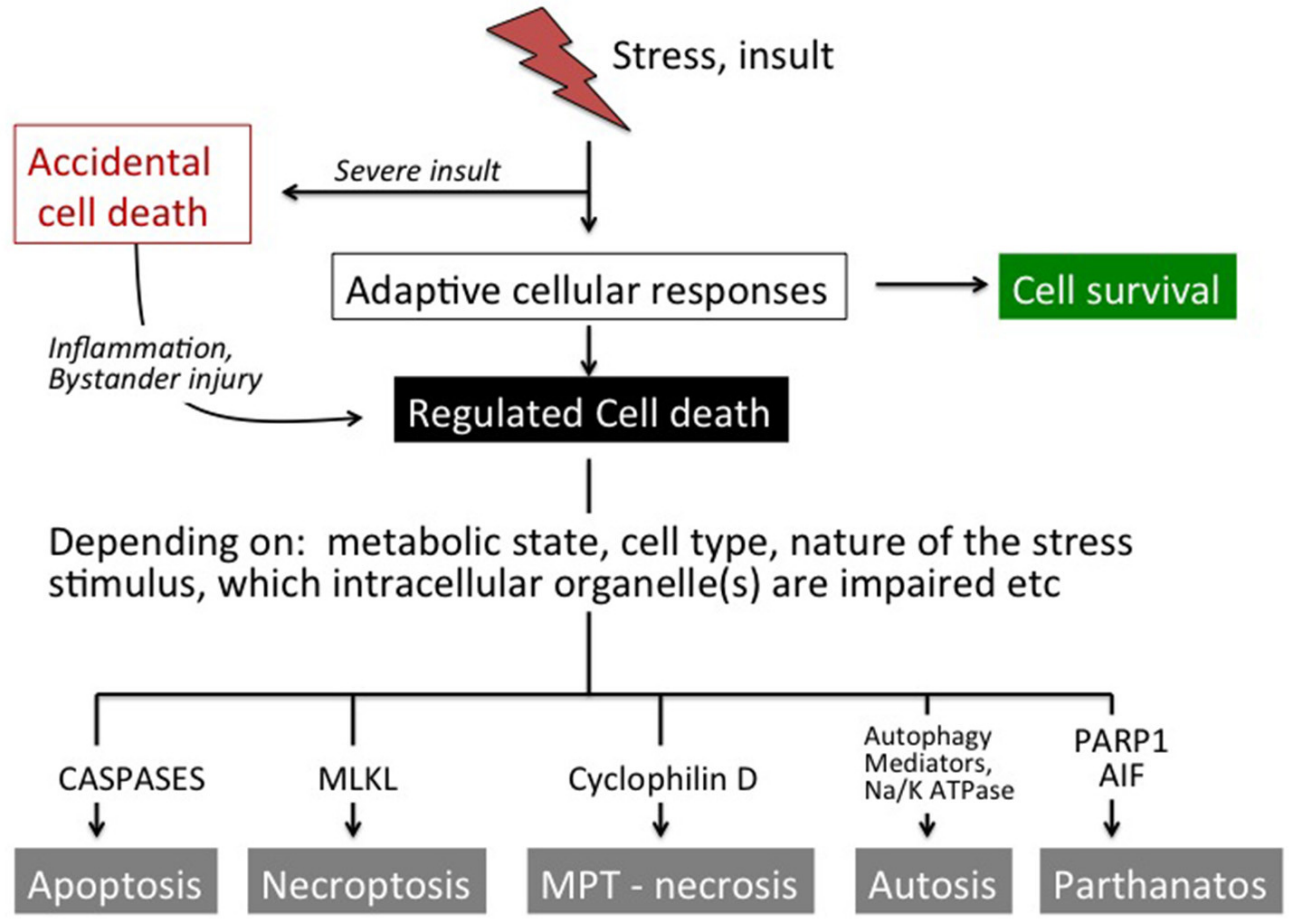
Overview of cell death pathways. In response to mild stress stimuli a number of compensatory mechanisms will be activated most often leading to cell survival. Moderate-to-severe insults may trigger regulated cell death. Depending on several factors such as the metabolic situation, cell type, nature of the stress stimulus and which intracellular organelle(s) that are affected, the cell undergoes apoptosis (caspase activation), necroptosis (MLKL activation), necrosis (via opening of the MPT pore), autophagic cell death (autophagy/ Na^+^ K^+^ ATPase) or parthanatos (PARP1, AIF). Severe insults cause accidental cell death that cannot be modulated genetically or by pharmacological means. However, accidental cell death leads to the release of factors (DAMPs) that initiate systemic effects as well as inflammation and (regulated) secondary brain injury in neighboring tissue.

Exposure of the brain to stress or an insult induces a number of adaptive responses that can culminate in the reestablishment of cellular homeostasis (Green et al., 2014; Vanden Berghe et al., 2014). However, when the stress is severe and/or the endogenous protective processes are not sufficiently effective to restore physiological functions the cell will die. Triggers of cell death can emanate from many organelles including the nucleus, mitochondrion, endoplasmic reticulum (ER), lysosomes, cytoskeleton, and/or plasma membrane, depending on the stress (Galluzzi et al., 2014). There are many alternative routes leading to cellular demise, such as necrosis/necroptosis, apoptosis, parthanatos, and autosis (Figure 1, Table 1) and the predominant mechanism will depend on metabolic state, severity and type of insult, cell type, developmental age and other factors (Kroemer et al., 2009; Green et al., 2014; Galluzzi et al., 2015). In some situations when one route is inhibited cell death may occur via a different route (Jouan-Lanhouet et al., 2012) and in many pathological situations mixed forms of morphological phenotypes are detected (Puka-Sundvall et al., 2000; Northington et al., 2001). Therefore, traditional morphology-based classifications (Table 1) may not always inform on the biochemical steps leading to cell death and hence what neuroprotective strategy may be successful (Galluzzi et al., 2015). The effect of genetic and/or pharmacological intervention on long-term functional cell recovery often provides more important information with regard to the essential components in a specific route of cell death.

**Table 1:** Comparison between morphological features of type I, type II, and type III cell death.

**Parameter**	**Apoptotic cell death**	**Autophagic cell death (autosis)**	**Necrotic cell death (including necroptosis)**
Plasma membrane	Preserved, blebbing	Rupture in late phase, sometimes blebbing	Rupture early
Nucleus	Compaction, pyknosis late: fragmentation (karyorrhexis)	Minor changes autosis: focal concavity, dilatation of perinuclear space	Dilatation of nuclear membrane
Chromatin	Margination, condensation	Minor/mild condensation	Mild-moderate condensation and clumping
Mitochondria	Normal	Mild dilatation, autosis: abnormal internal structure late: depletion	Swelling
Cytoplasm	Shrinkage	Vacuolization, i.e., numerous autophagosomes and Autolysosomes; autosis: ER fragmentation and depletion	Minor
Other	Rounding of cells and detachment from surface, apoptotic bodies including fragments of chromatin, and preserved organelles	Autosis: membrane bound densities in perinuclear space, increased cell surface adhesion	Cell and organelle swelling

*Summary based on Kerr et al. (1972), Kerr et al. (1994), Savitz and Rosenbaum (1998), Liu and Levine (2015), Leist and Jäättelä (2001), Galluzzi et al. (2015)*.

Cell death can also be classified into *accidental* and *regulated* (Figure 1; Galluzzi et al., 2015). Accidental cell death is evoked by severe insults (such as severe trauma, core of an ischemic infarct), which causes immediate cellular demise that does not involve a specific molecular mechanism and cannot be prevented or modulated (Green and Kroemer, 2005). However, cells undergoing accidental cell death release products (damage-associated molecular patterns; DAMPs) that often have direct toxic effects on surrounding cells that survived the initial insult and may extend the primary injury (Vanden Berghe et al., 2014; Galluzzi et al., 2015). DAMPs also have immunogenic properties and contribute to an inflammatory response that may exert injury and aggravate the situation further (Zhang et al., 2010; Vanden Berghe et al., 2014). Various interventions that attenuate DAMP-induced cellular actions can provide protective effects (Zitvogel et al., 2010). So even if accidental cell death cannot be targeted directly, its consequences can be intercepted and bystander injury prevented to some extent. On the contrary, regulated death (not to be confused with the term programmed cell death which is used synonymously with apoptosis) involves the molecular machinery of the cell (Figure 1) and its course can indeed be modulated by pharmacological and genetic means (Kroemer et al., 2009; Galluzzi et al., 2014, 2015). Regulated cell death usually occurs with some delay in situations when endogenous protective mechanisms fail to restore cellular homeostasis.

In the developing brain, cell damage can be induced by a variety of insults, such as hypoxia (Schwartz et al., 2004), hyperoxia (Reich et al., 2016), hypoxia-ischemia (Rice et al., 1981), trauma (Bittigau et al., 2004), and inflammation/infections (Strunk et al., 2014). However, most knowledge on mechanisms of cell death emanates from studies *in vivo* and *in vitro* in models of hypoxia-ischemia so therefore we will focus mostly on that work.

HI results in an initial depletion of high energy phosphates, in particular ATP and phosphocreatine. These levels return transiently to baseline but are followed by a second more prolonged depletion of cellular energy reserves accompanied by progression of brain injury (Blumberg et al., 1997; Hagberg et al., 2014). These disturbances in energy metabolism trigger a number of pathophysiological responses that ultimately lead to cell death. Previous studies show that HI in the immature brain can induce apoptosis (Edwards et al., 1997; Zhu et al., 2000; Northington et al., 2001), necroptosis/necrosis (Northington et al., 2011; Galluzzi et al., 2012a) as well as autophagic cell death/autosis (Koike et al., 2008; Ginet et al., 2009; Liu et al., 2013).

Mitochondria are involved in adaptive and metabolic responses to injury, as well as in most forms of cell death including apoptosis (intrinsic and to some degree extrinsic pathway), regulated necrosis (not always essential), parthanatos and autophagic cell death (Rosenberg et al., 1989; Yager et al., 1996; Galluzzi et al., 2012a,b, 2015; Thornton et al., 2012; Vanden Berghe et al., 2014). Notably, mitochondria have a key role in the initiation and execution of cell death also in the immature brain (Chavez-Valdez et al., 2012; Hagberg et al., 2014). In this review we will briefly update basic knowledge of the different forms of regulated cell death and then summarize morphological and biochemical evidence for apoptotic, necrotic/necroptotic and autotic cell death in immature brain exposed to HI.

## Apoptotic cell death

### The apoptotic cell machinery

**Figure 2:**
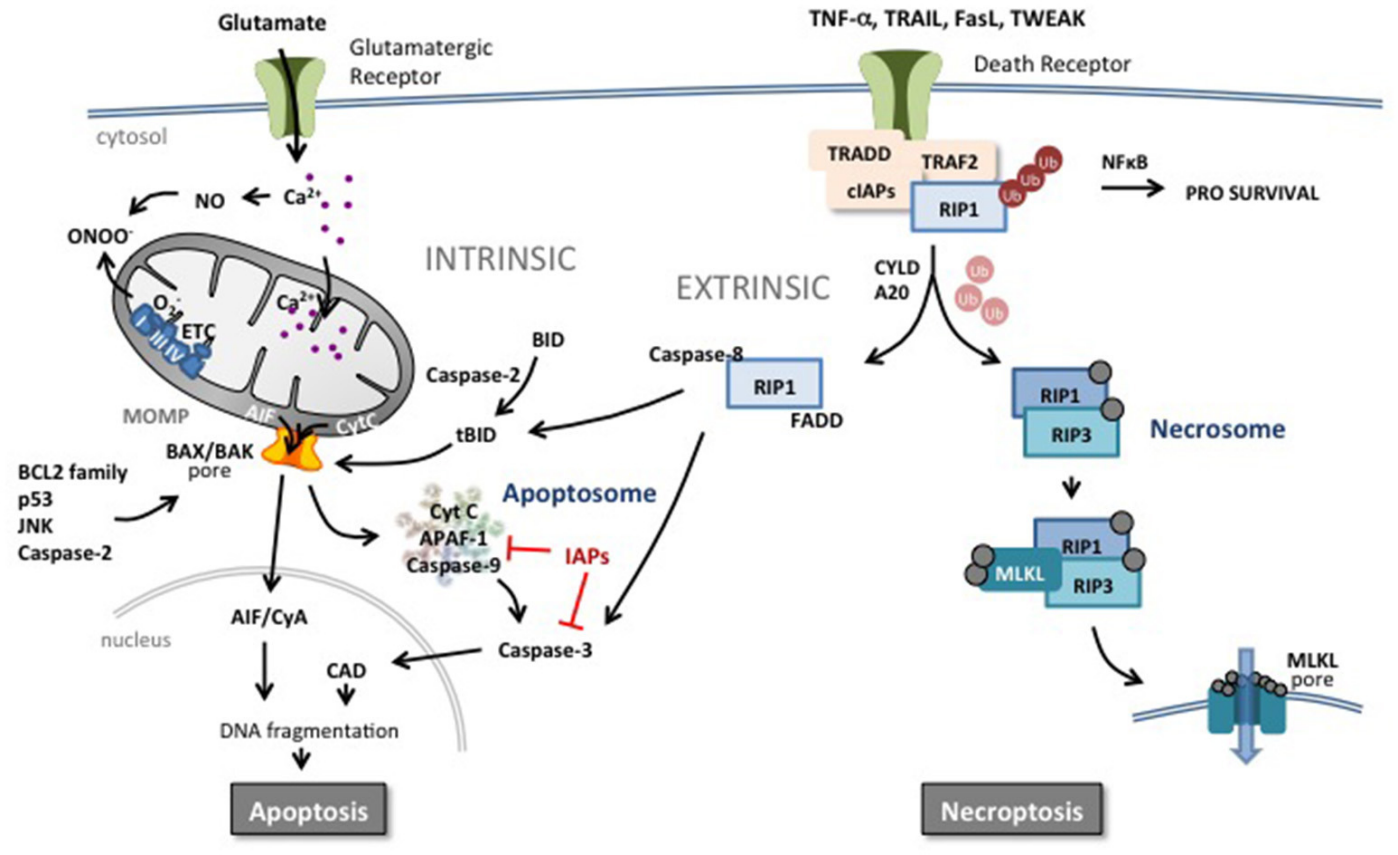
Apoptotic and necroptotic mechanisms. The intrinsic pathway is triggered by mitochondrial impairment related to glutamate overflow leading to excessive intracellular Ca^2+^ accumulation and accumulation of NO and ROS. Such intramitochondrial alterations can trigger a shift in localization of pro-apoptotic proteins such as cytochrome C (CytC) from the inner mitochondrial membrane to the intermembrane space. In addition, perturbation in the nucleus, endoplasmic reticulum or in other organelles can increase the pro- vs. anti-apoptotic BCL2 protein family balance, JNK, caspase-2 activity or p53 expression at the level of the mitochondrial outer membrane. Such changes trigger mitochondrial outer membrane permeabilization (MOMP) and release of pro-apoptotic proteins into the cytosol. Cyt C initiates the assembly of the apoptosome leading to the activation of caspase-9 and subsequently the executioner caspase-3 and DNA cleavage through activation of caspase-activated DNase (CAD). Inhibitors of apoptosis (IAPs) block the apoptosome and caspase activity. Apoptosis-inducing factor (AIF) binds to cyclophilin A and the complex translocates to the nucleus and triggers chromatinolysis. Brain injury including HI results also in an increase of circulating death receptor ligands such as TNF-α, Fas, TRAIL etc. In response to ligand-receptor binding, complex I is formed at the membrane comprising the receptor, adaptor protein and RIP1 which is rapidly polyubiquinated (Ub) by cIAP. This complex can trigger the NFκB pathway and a prosurvival response. However, deubiquinating enzymes and Smac (which degrades cIAPs) release RIP1 and commit the cell to a cell death pathway. In the presence of caspases, RIP1 forms a complex with active caspase-8 and FADD, triggering the extrinsic apoptotic pathway. Caspase-8 can directly trigger executioner caspase-3 or cleave and activate BID (forming truncated BID, tBID) which can trigger MOMP. Caspase-8 can also prevent the induction of necroptosis by cleaving key proteins. In the absence of caspases, RIP1 interacts with RIP3 which autophosphorylates and subsequently recruits MLKL to the necrosome complex. Phosphorylated MLKL will target the necrosome to membrane lipid-rich regions such as mitochondrial or plasma membranes, forming pores allowing influx of ions and cell swelling.

Apoptosis can be triggered by intracellular (intrinsic) and extracellular (extrinsic) stimuli (Figure 2; Kerr et al., 1972, 1994). The **intrinsic** pathway relies on mitochondrial outer membrane permeabilization (MOMP) resulting in the release of a number of pro-apoptotic proteins into the cytosol including holocytochrome *c* (Cyt c), apoptosis-inducing factor (AIF), second mitochondria-derived activator of caspases (SMAC) and endonuclease G (EndoG) (Hengartner and Horvitz, 1994; Wei et al., 2001; Ravagnan et al., 2002; Galluzzi et al., 2012a,b). Cyt c will form a complex (apoptosome) with deoxy-ATP, apoptotic peptidase-activating factor 1 (APAF-1) and caspase-9 leading to the downstream activation of the executioner caspase-3 (Li et al., 1997; Bratton and Salvesen, 2010; Galluzzi et al., 2012a). MOMP depends on two pore-forming pro-apoptotic members of the B-cell lymphoma 2 (BCL2) family, Bcl-2-associated X protein (BAX) and Bcl-2-antagonist/killer 1 (BAK1) (Figure 2). The opening of the BAX/BAK1 pore is regulated by anti-apoptotic BCL2 family proteins such as BCL2 itself, BCL2 like 1 (BCL-X_L_), and myeloid cell leukemia 1 (MCL1) and the pro-apoptotic members BCL2 binding component 3 (also known as PUMA), BCL2-like 11 (known as BIM) and BH3- interacting domain death agonist (BID)(Moldoveanu et al., 2014). The activity of MOMP is also controlled by p53, c-jun N-terminal kinase (JNK) and caspase-2 (Galluzzi et al., 2014; Baburamani et al., 2017).

In the **extrinsic** pathway, binding ligands to a death receptor leads to activation of caspase-8. Approximately 20 ligand-receptor pairings are now included in the death receptor ligand tumor necrosis factor (TNF) superfamily (Pennica et al., 1984; Vanden Berghe et al., 2014). These TNF-receptor and TNF-receptor-like molecules are similar in structure to TNF and function as trimers (both ligands and receptors) (Pennica et al., 1984). Because of the similarity of their structure, multiple ligands are able to bind and induce signaling through one receptor, or a single ligand is able to bind multiple receptors. Some of the receptors contain the so-called death domain in their intracellular domain (e.g., TNF-R1, DR4, DR5, Fas) and are able to trigger apoptosis when activated from the binding of the corresponding ligand (e.g., TNF-α, TRAIL, FasL) (Holler et al., 2000). This extrinsic pathway of apoptosis continues with the activation of a death-inducing signaling complex (DISC) adjacent to the death domain of the receptor. Activated DISC catalyzes the proteolytic cleavage and activation of procaspase-8 (Love, 2003; Vanden Berghe et al., 2014; Figure 2). Activated caspase-8 either directly activates caspase-3 or mediates cleavage of BID to truncated BID (tBID), which integrates different death pathways at the mitochondria. tBID translocates to mitochondria where it interacts with other proapoptotic proteins and triggers the release of apoptogenic factors leading to caspase-dependent and caspase-independent cell death. Death receptors can also trigger necroptosis especially under conditions when caspase-8 is inactive (Vanden Berghe et al., 2014) (see section below on regulated necrosis).

### Apoptosis in the immature brain

Apoptosis is critical for brain development and determines the size and shape of the central nervous system (Kuan et al., 2000). In some regions more than half of neurons initially formed undergo apoptotic cell death (Raff et al., 1993). Many of the proteins involved in apoptosis such as caspase-3 (Blomgren et al., 2001), APAF1 (Ota et al., 2002), and BCL2 -family proteins (Merry et al., 1994; Vekrellis et al., 1997; Soane et al., 2008) are upregulated during brain development. Mice devoid of caspase-3 (Kuida et al., 1996) or caspase-9 (Kuida et al., 1998) exhibit hyperplastic disorganized brains (whereas other organs like the thymus with ongoing apoptosis develop normally) supporting the concept that caspases are of particular importance in shaping the developing brain. Thus, several components of the intrinsic pathway are markedly upregulated in the postnatal brain because of ongoing physiological apoptosis as part of CNS development.

### Role of the intrinsic pathway in perinatal brain injury

Mitochondria in the developing brain are prone to permeabilization in response to HI (Northington et al., 2001; Wang et al., 2001, 2004). Proapoptotic proteins (e.g., Cyt C and apoptosis inducing factor, AIF) are released from mitochondria, the apoptosome forms, and downstream executioner caspases (particularly caspase-3) are activated after hypoxic–ischemic insult (Cheng et al., 1998; Wang et al., 2001; Sugawara et al., 2004). Pathways dependent on AIF (Zhu et al., 2003, 2007a,b) and caspases seem to be more strongly activated in the immature brain than in the adult brain (Hu et al., 2000; Zhu et al., 2005), and mitochondrial permeabilization has been proposed to mark the point of no return in hypoxic–ischemic injury of the immature brain (Hagberg, 2004; Galluzzi et al., 2009).

The molecular mechanisms of mitochondrial permeabilization under these conditions are still not completely understood. Mitochondria can permeabilize through either BAX–BAK-dependent pore formation or opening of the mitochondrial permeability transition pore (MPT-pore) (Galluzzi et al., 2009; Rasola et al., 2010). The MPT-pore is dependent on cyclophilin D, and is formed when both the inner and outer leaflets of the mitochondrion are at their closest points (Rasola et al., 2010). The molecular identity of the MPT-pore is still lacking but recent studies suggest that ATP synthase is an important component (Giorgio et al., 2013; Bonora and Pinton, 2014; Gerle, 2016). Permeabilization of the inner mitochondrial membrane results in leakage of solutes, depolarization due to equilibration of the proton gradient, and swelling of the mitochondrion due to disruption of the outer membrane. Cell death mediated by the MPT-pore (in contrast with BAX-mediated permeabilization, below) is predominantly necrotic (through Ca^2+^ imbalance and bioenergetic failure) and facilitates development of adult brain ischemic injury, because deficiency of the cyclophilin D gene *Ppid* and cyclophilin D inhibitors are neuroprotective (Kuroda et al., 1999; Schinzel et al., 2005). However, in the immature brain, cyclophilin D gene (*Ppid*) deficiency aggravates rather than lessens hypoxic–ischemic injury, and cyclophilin D inhibitors do not reduce injury (Puka-Sundvall et al., 2001; Wang et al., 2009). Instead, BAX-inhibitory peptides (Wang et al., 2009, 2010; Sun et al., 2015) and BAX deficiency (Gibson et al., 2001) substantially protect the immature brain in mice, suggesting that BAX-dependent permeabilization of the outer membrane (rather than cyclophilin-D-mediated opening of the MPT-pore) is critical in the developing brain and results in apoptotic cell death. Furthermore, studies which ablate the effects of BAX-mediated mitochondrial membrane permeabilization (e.g., knockout models of BIM and BAD (Ness et al., 2006), Tat-BCL-xL (Yin et al., 2006), Bcl-xL transgenic mice (Parsadanian et al., 1998) all exhibit reduced brain injury after neonatal HI). Interestingly, BCL-xL seems to reduce primarily the delayed apoptotic cell death rather than the early (necrotic) loss of cells (Dietz et al., 2007). In rats subjected to neonatal HI, there is a peak of caspase-3 activity observed 24 h after the insult (Cheng et al., 1998) which remains elevated for a significant number of days (Wang et al., 2001). Caspase inhibitors have been shown to be neuroprotective in immature models of hypoxia-ischemia (Cheng et al., 1998; Zhu et al., 2007a,b).

AIF can also translocate to the mitochondrial intermembrane space in response to oxidative stress, induction of poly (ADP-ribose) polymerase (PARP) 1, and activation of proteases (e.g., calpains or cathepsins). This translocation is necessary for the subsequent relocation of AIF to the nucleus after MOMP (Modjtahedi et al., 2006; Krantic et al., 2007). Indeed, AIF does translocate to the nucleus after neonatal HI (Zhu et al., 2003) and mice with lower expression of AIF are less vulnerable to HI especially in combination with administration of a caspase inhibitor (Zhu et al., 2007b) suggesting that mitochondrial AIF release contributes to brain injury in such situations. AIF binds to cyclophilin A in the cytosol, the protein complex translocates to the nucleus and induces non-caspase dependent chromatinolysis (Zhu et al., 2007a). This specific route of cell death that depends on PARP-1 and AIF is often referred to as *Parthanatos* (Figure 1) rather than apoptosis (Fatokun et al., 2014) and exhibits morphologic features of regulated necrosis rather than apoptosis (Vanden Berghe et al., 2014). The protein IDUNA has been discovered to inhibit this pathway in the adult brain (Andrabi et al., 2011) which seems to apply also to the immature brain (Yang et al., 2017). Taken together, these data suggest that BAX-dependent MOMP is a critical event in delayed brain injury in the immature brain because it leads to both activation of caspase-dependent and caspase-independent cell death.

#### Upstream regulators of MOMP and apoptosis

##### Excitotoxicity

Excitotoxicity involves the accumulation of extracellular excitatory amino acids, such as glutamate leading to activation of NMDA and AMPA receptors which in turn trigger influx of calcium and sodium into the cell (Johnston, 2005). The subsequent increase of intracellular calcium elicits production of NO as well as reactive oxidative species which contributes to mitochondrial perturbation and MOMP leading to apoptotic cell death (Figure 2; Hagberg et al., 2014).

##### p53

p53 is a tumor suppressor that triggers apoptosis via multiple pathways including cell cycle arrest and the regulation of autophagy through transactivating proapoptotic and repressing antiapoptotic genes (Morrison et al., 2003; Green and Kroemer, 2009). It is highly conserved and regulates cell death resulting from a wide variety of both physiological and pathological stimuli. p53 also has cytoplasmic actions at the mitochondrial level and can promote BAX-dependent mitochondrial permeabilization (Green and Kroemer, 2009). In unstressed neurons, p53 expression is generally low, limited by its association with its negative regulator MDM2 which functions as a ubiquitin ligase, targeting polyubiquitinated p53 for degradation (Honda et al., 1997). Cellular stress displaces p53 from MDM2, and subsequently p53 expression is stabilized through substantial posttranslational modification (Morrison et al., 2003). The classical role for p53 is as an activator of transcription, and, on stabilization, it accumulates in the nucleus where it upregulates the transcription of proapoptotic genes such as PUMA, BAX, and NOXA (Riley et al., 2008). More recently a transcription-independent role was described in which activated p53 accumulates in the cytosol where it is sequestered by the antiapoptotic BCL2 proteins for example, BCL-X_L_ (Green and Kroemer, 2009). However, increased PUMA expression mediated by nuclear p53 displaces BCL-X_L_ allowing p53 to activate BAX, promoting its oligomerization, mitochondrial outer membrane permeabilization, and inducing apoptosis (Chipuk et al., 2005; Green and Kroemer, 2009). Indeed, p53 is upregulated and accumulates in the nucleus and mitochondria in an *in vivo* rat model of neonatal HI (Nijboer et al., 2008a,b). In consequence, there is an upregulation of apoptotic pathways leading to activation of caspase-3. The authors identified a pathway involving NFκB upstream of p53 and were able to decrease p53 accumulation (thus increasing neuronal survival), in response to neonatal HI by treating with the NFκB inhibitor peptide (Nijboer et al., 2008a,b; Van Der Kooij et al., 2010). Furthermore, pifithrin-μ (an inhibitor of mitochondrial p53; Strom et al., 2006) administered after neonatal HI in rats provided significant protection with a 6 h therapeutic window (Nijboer et al., 2011), supporting that the p53-BAX dependent pathway is important in HI brain injury. However, we recently found that p53 gene deficiency only provided partial protection in the posterior part of the brain in response to moderate HI (Baburamani et al., 2017) and we suspect that the protective effect of pifithrin-μ may relate to factors independent of p53, such as heat shock proteins and inflammation (Leu et al., 2009; Fleiss et al., 2015).

##### c-Jun N-terminal Kinases (JNKs)

c-Jun N-terminal Kinases (JNKs) are members of the mitogen-activated protein kinase (MAPK) family and, as such, are activated in response to stress. There are three mammalian junk genes and 10 expressed isoforms as the result of alternative splicing; however, it is JNK3 that is predominantly active in the brain (Dreskin et al., 2001). In a mouse model in which JNK3 expression is ablated, both adult and neonatal animals were partially protected against HI insult, and, in newborn animals, levels of c-jun were reduced compared with wild-type animals (Kuan et al., 2003; Pirianov et al., 2007). Pharmacological inhibition of JNK (either by TAT-JBD or D-JNKi) in neonatal mice after HI resulted in reduced infarct size, preservation of mitochondrial integrity and a more favorable behavioral outcome (Nijboer et al., 2013). This correlates with an earlier study suggesting that expression of c-Jun and its subsequent phosphorylation was increased on ischemic injury (Herdegen et al., 1998). JNK3 is hypothesized to act upstream of the proapoptotic BCL2 family as JNK3-mediated increases in BIM and PUMA expression were absent in JNK3 gene knock-out mice (Pirianov et al., 2007). Furthermore, Forkhead transcriptional factor (FOXO3a), a critical effector in JNK activation, is probably also involved in the pro-apoptotic effect of JNK activation as JNK inhibition prevents FOXO3a translocation to the nucleus in the immature brain after HI (Li et al., 2015). In addition, activation of caspase-3 was also decreased suggesting that activation of JNK3 in response to hypoxic-ischemic insult results in caspase-dependent apoptosis. The importance of JNK is further supported by a recent study showing that inhibition of Apoptosis signal-regulating kinase 1 (ASK1) confers protection in HI. ASK1 activates JNK and prevented phosphorylation of JNK, TUNEL expression, and caspase-3 activation in a neonatal model of HI (Hao et al., 2016).

##### Caspase-2

Caspase-2 is a member of the initiator subgroup of caspases and is developmentally regulated (Kumar et al., 1994). Activation of caspase-2 is dependent on its dimerization and subsequent cleavage which is facilitated through interaction with p53-induced death domain-containing protein (PIDD) and RIP associated ICH-1/CED3 homologous protein with a death domain (RAIDD) (Duan and Dixit, 1997; Baliga et al., 2004; Tinel and Tschopp, 2004) in some cellular systems. In addition, caspase-2 can be triggered by nuclear DNA damage, endoplasmic reticulum or Golgi stress via a mechanism not dependent on PIDD/RAIDD (Galluzzi et al., 2014). Once activated, caspase-2 promotes BID cleavage resulting in BAX translocation and release of Cyt C (Lassus et al., 2002). Notably, neonatal caspase-2 null mice are partially protected from excitotoxic and HI injury (Carlsson et al., 2011), in contrast with adult caspase-2 knockout mice (Bergeron et al., 1998). A high expression of caspase-2 was found in neonatal mice, rats and in postmortem human tissue from neonates (Carlsson et al., 2011). Interestingly, a group II caspase inhibitor, TRP601, has been developed which targets caspase-2 and caspase-3. Neonatal animals subjected to excitotoxicity, arterial stroke or HI were significantly protected against white and gray matter loss (Chauvier et al., 2011).

##### Cyclin-dependent kinase 5 (CDK5)

Cyclin-dependent kinase 5 (CDK5) belongs to a group of serine/threonine kinases that takes part in the regulation of the cell cycle under normal conditions. However, during pathological situations, p35 is cleaved by calpains to generate p25 which overactivates CDK5 leading to phosphorylation and dysregulation of axonal TAU proteins and glucocorticoid receptors enhancing apoptotic cell death. Inhibition of p25/CDK5 before or after neonatal HI attenuates caspase-3 activation (Tan et al., 2015), brain injury and improves neurological outcome in neonatal rats suggesting that CDK5 is another potential trigger of apoptotic cell death.

##### PTEN/AKT/GSK3β/foxo3a

PTEN/AKT/GSK3β/foxo3a pathway seems critical in the induction of apoptosis in the neonatal brain. Phosphatase and tensin homolog deleted on chromosome 10 (PTEN) antagonizes phosphatidylinositol-3-kinase-AKT signaling. Inhibition of PTEN has been shown to increase pAKT, decrease FOXO3a translocation to the nucleus and downregulate BIM and apoptotic cell death in the neonatal brain after HI (Zhao et al., 2013). Phosphorylation of Akt also inhibits the activity of glycogen synthase kinase-3β (GSK-3β), which triggers caspase-3 dependent apoptosis and the GSK-3β inhibitor Tideglusib has been shown to reduce caspase-3 and -9 activation as well as reduce HI brain injury (Wang et al., 2016). Furthermore, neuroprotection by progesterone, insulin-like growth factor, growth hormone and its analog Hexarelin all seems to be related to activation of Akt and inhibition of GSK3β (Gustafson et al., 1999; Brywe et al., 2005a,b; Li et al., 2014) and reduction of caspase-3 dependent apoptosis.

#### Timing of MOMP

The timing of mitochondrial permeabilization is debated, but most study findings suggest that it happens 3–24 h after hypoxia–ischemia—i.e., starting during the latent phase and proceeding into the secondary phase of injury depending on severity of insult, animal model, and brain region (Cheng et al., 1998; Northington et al., 2001; Gill et al., 2002; Wang et al., 2004; Zhu et al., 2005; Hagberg et al., 2014). These proposed timings are also supported by evidence from interventions that block mitochondrial permeabilization, which are effective if given up to 6 h after hypoxia–ischemia (Wang et al., 2010; Chauvier et al., 2011; Nijboer et al., 2011, 2013).

### Extrinsic pathway and death receptors in perinatal brain injury

During inflammation initiated by perinatal brain injury (Hagberg et al., 2012), activation of intrinsic and extrinsic immune cells will produce reactive oxygen species, release excitatory amino acid agonists, proinflammatory cytokines (e.g., IL-1β, IL-18, TNF-α), chemokines (Bona et al., 1999), and tumor necrosis factors (e.g., TNF-α, TNF-β, FasL, TRAIL, TWEAK) (Taylor et al., 2005; Yepes et al., 2005; Hoffmann et al., 2009; Hagberg et al., 2015) that may contribute to cell death.

TNF-α activity is mediated through activation of two receptors: low-affinity TNFR1 (p55) and the high-affinity TNFR2 (p75) (Tartaglia et al., 1991), found in both neuronal (Dziewulska and Mossakowski, 2003; Figiel and Dzwonek, 2007) and glial cell populations (Dopp et al., 1997). Although the extracellular domains of both receptors have a high degree of homology, their intracellular domains differ significantly (Dembic et al., 1990; Vanden Berghe et al., 2014). This leads to complex signal transduction pathways that can be triggered and may result in activation of the antagonistic functions of these two receptors (Tartaglia et al., 1991; Marchetti et al., 2004). When activated, the intracellular part of TNFR1 containing the death domain triggers apoptosis (Hsu et al., 1995), whereas TNFR2 lacks that domain—its activation triggers neuroprotection through activation of NFκB (Song et al., 1997). There are several pieces of evidence that suggest the involvement of the TNF pathway in the development of white matter damage. Children who develop cerebral palsy show increased blood levels of TNF-α (Nelson et al., 1998), and TNFR1 is critical for LPS-mediated sensitization to oxygen/glucose deprivation *in vitro* (Markus et al., 2009). Moreover, deletion of the TNF gene cluster abolishes LPS-mediated sensitization of the neonatal brain to HI insult (Kendall et al., 2011). TNF-α treatment appears to be toxic for oligodendroglial precursor cells (OPCs) (Yu et al., 2000) and potentiates the IFN-γ toxicity on those cells *in vitro* (Andrews et al., 1998). TNF is also implicated in brain neuroprotection. It has been demonstrated that neuronal damage after ischemic and excitotoxic insults are enhanced in TNFR KO mice (Bruce et al., 1996). The neuroprotective role for TNF in cerebral ischemia is at least partly attributed to TNFR2 activity (Lambertsen et al., 2009).

FasL is able to bind with Fas death receptor triggering apoptosis and with Decoy receptor 3 (Pitti et al., 1998). HI activates Fas death receptor signaling in the neonatal brain (Felderhoff-Mueser et al., 2000) and HI brain injury is reduced in mice lacking Fas death receptors (Graham et al., 2004). It is shown that Fas expression in primary OPCs is higher than in mature oligodendrocytes (Andrews et al., 1998), implying higher susceptibility to FasL at earlier developmental stages.

Two TRAIL receptors in humans contain cytoplasmic death domains (DR4 and DR5) and have the capacity to induce apoptotic cell death (Pan et al., 1997; Walczak et al., 1997), whereas Decoy receptor 1 and Decoy receptor 2 lack functional death domains and thus are considered to act as decoy receptors (Marsters et al., 1997; Sheridan et al., 1997). In mice, two membrane decoy receptors mDcTRAILR1 and mDcTRAILR2 have been reported (Schneider et al., 2003), and only one death-mediating TRAIL receptor, which has the highest homology with the human TRAIL receptor DR5 (Wu et al., 1999). Using a neonatal mouse model we recently found that the expression of TRAIL, DR5, and mDcTRAILR2 was significantly increased after HI (Kichev et al., 2014). TRAIL protein was expressed primarily in microglia and astroglia, whereas DR5 co-localized with neurons and oligodendroglial precursors *in vivo*. Recombinant TRAIL exerted toxicity alone or in combination with oxygen glucose deprivation and TNF-α/IFN-γ exposure in primary neurons suggesting that the elevated TRAIL levels after HI may aggravate brain injury during the recovery phase (Kichev et al., 2014). This assumption is supported by studies showing that injection of soluble DR5 receptor significantly reduces infarct volume after ischemia at least in adult rodent models (Cui et al., 2010).

Only one receptor for TWEAK has been identified so far in both humans and rodents, the fibroblast growth factor inducible 14 (Fn14) (Wiley et al., 2001). The Fn14 cytoplasmic tail does not contain a canonical death domain, and TWEAK binding to Fn14 can induce multiple cell death pathways in different cellular contexts (Potrovita et al., 2004; Cannella et al., 2007). Intracerebroventricular injection of soluble Fn14 (Yepes et al., 2005) reduces significantly the infarct volume after ischemia in adult rodent models but its role in immature brain injury is unknown.

### Necrosis and necroptosis

The concept of necrosis as a form of cell death is long-standing, first mentioned in 1859 in Virchow's textbook on Cellular Pathology (Majno and Joris, 1995). Necrosis is defined as rapid, accidental or uncontrolled cell death characterized by cell swelling and membrane rupture leading to an inflammatory response (Laster et al., 1988; Lu et al., 2014; Table 1). After insult, an initial depletion of ATP disrupts the action of plasma membrane transporters such as Na^+^, K^+^ ATPase causing an influx of Na^+^ and Cl^−^ accompanied by increases in intracellular Ca^2+^ and water (Fiers et al., 1999). The subsequent increase in intracellular volume ultimately results in plasma membrane collapse and the release of cell contents into the extracellular space triggering the host's inflammatory response caused by exposure to DAMPs, such as mitochondrial DNA (Scaffidi et al., 2002; Zhang et al., 2010; Figure 1).

However, within the last three decades, this view of passive necrosis has been challenged by the discovery that in response to ligands such as TNF family cytokines, regulated cell death was triggered with a morphology resembling that of necrosis (plasma membrane breach, mitochondrial swelling) (Ofengeim and Yuan, 2013). Necroptosis or programmed necrosis (Laster et al., 1988; Galluzzi et al., 2011, 2012c) is a form of highly regulated cell death that occurs in an environment that is either dramatically depleted of ATP (Leist et al., 1997; Nicotera et al., 1998) or in which caspases are inhibited (Vercammen et al., 1998; Cho et al., 2009; Kaiser et al., 2011).

### The cellular mechanism of necroptosis

In common with the extrinsic pathway of apoptosis, necroptosis is commonly induced by death receptor ligands such as TNF-α, Fas, TRAIL (Figure 2), or by Toll-like receptor (TLR) 3 and 4 signaling (Vanlangenakker et al., 2012). Binding of the ligand to the TNF receptor initiates the assembly of a plasma membrane-associated complex (Figure 2) into which the adaptor protein TRADD and receptor-interacting kinase 1 (RIP1 also known as RIPK1) are recruited by virtue of common death domains (Stanger et al., 1995; Hsu et al., 1996; Hitomi et al., 2008). The complex is further stabilized by the recruitment of cellular inhibitor of apoptosis proteins (cIAPs, Bertrand et al., 2008). However, RIP1 can initiate numerous signaling pathways including pro-survival NF-κB and MAPK activation (Ting et al., 1996; Bertrand et al., 2008). How, then, is its signaling diverted to the induction of cell death? The answer lies in the ubiquitination state of RIP1. Rapid polyubiquitination of RIP1 by cIAPs occurs as the DISC complex forms at the membrane, and pushes RIP1 function toward NF-κB activation and MAPK signaling (Bertrand et al., 2008). However, degradation of cIAPs by autoubiquitination (assisted by the action of SMAC, Du et al., 2000) and deubiquitination of RIP1 by deubiquinating enzymes Cylindromatosis (CYLD) and A20 results in release of RIP1 from the complex (Wertz et al., 2004; Moquin et al., 2013). This marks the point at which the cell commits to a cell death outcome, but even here, RIP1 signaling can still be diverted from necroptosis to the induction of apoptosis if caspase-8 is present in the cell (Wang et al., 2008). RIP1 can form a complex with Fas-associated death domain (FADD) and caspase-8 initiating the latter's conversion to its active form and subsequently triggering apoptosis (Wang et al., 2008; Remijsen et al., 2014; Figure 2).

Caspase-8 actively inhibits necroptosis through degradation of RIP1 and RIP3 (Lin et al., 1999; Oberst et al., 2011) but in the absence of caspase-8, viral or genetic inhibition (Cho et al., 2009; Kaiser et al., 2011) or high RIP3 expression (Zhang et al., 2009), necroptosis will occur. RIP1 and RIP3 interact through their RHIM (RIP homotypic interaction motif) domains resulting in the formation of the necrosome, a fibrillar, amyloid-like structure (Li et al., 2012) and further recruitment of RIP3 to the necrosome occurs (Wu et al., 2014). RIP3 autophosphorylates (Ser 227), and recruits its substrate pseudokinase mixed lineage kinase domain-like (MLKL) into the necrosome where it is phosphorylated by RIP3 at Thr 357 and Ser 358. Phosphorylation and activation of MLKL results in its oligomerization (Wang et al., 2014) and in this form it can bind membrane lipids such as phosphotidylinositol phosphate or the mitochondrial-located cardiolipin (Dondelinger et al., 2014). These activated necrosomes orchestrate the permeabilization of both cell and organelle membranes and likely facilitate the cataclysmic membrane lysis observed in the execution of necrosis (Chen et al., 2014).

Necroptosis can also be induced by alternative routes. In the absence of caspase-8 or if FADD is inhibited by phosphorylation, interferons can transcriptionally upregulate the expression of the RNA-responsive protein kinase, which is capable of interacting with RIP1, subsequently promoting formation of the RIP1-RIP3 necrosome (Thapa et al., 2013; Mccomb et al., 2014). Interestingly, as with the role of RIP1 in NF-kB signaling, RIP1 acts as a scaffolding molecule as its kinase activity is dispensable for interferon-mediated necroptosis. TLR3 and TLR4 activation by LPS and dsRNA can trigger necroptosis in the absence of RIP1; instead, the RHIM-domain-containing protein TRIF interacts with RIP3 to recruit MLKL to the necrosome (He et al., 2011; Kaiser et al., 2013). Infection by murine cytomegalovirus can also trigger interaction between the RHIM domain protein DNA-dependent activator of interferon regulatory factors and RIP3 resulting in virus-induced necroptosis (Upton et al., 2012).

The presence of RIP3 and MLKL is pivotal for the execution of necroptosis, and it is worth remembering that only RIP3 and MLKL are true markers of necroptosis as RIP1 can participate in both prosurvival and apoptotic mechanisms as well as negatively regulating necroptosis itself by inhibiting spontaneous RIP3 activation (Orozco et al., 2014).

### Negative regulation of necroptosis

As can be inferred from above, there are a number of stages at which necroptosis can be inhibited, both by endogenous events and by addition of pharmacological reagents. The formation of the necrosome relies on the removal of ubiquitin from RIP1 and therefore upregulation of cIAPs or downregulation of Smads will prevent complex formation (Geserick et al., 2009). Necroptosis and apoptosis are fundamentally linked as certain ligands can trigger both pathways. In this situation, caspase-8 activation state sits at the divergence point through its degradation of RIP1 and RIP3; interestingly this negative regulation implies that necroptosis cannot truly be considered a caspase-independent form of cell death. Contributing to the prolonged ubiquitination of RIP1, CYLD is a substrate for cleavage by active caspase-8 which can also cleave RIP1 and RIP3 and therefore necroptosis is inhibited (Feng et al., 2007). In addition, caspase-8 homodimers promote apoptosis whereas caspase-8-FLIP heterodimers actively inhibit necroptosis (O'Donnell et al., 2011). During the search for substrates of RIP3, a small molecule inhibitor necrosulfonamide was identified which targets MLKL preventing formation of the necrosome (Sun et al., 2012). A chemical inhibitor of RIP1, necrostatin, and its derivatives (Degterev et al., 2008) has also been instrumental in dissecting the necroptosis pathway but as with many pharmacological compounds, care should be taken in the interpretation of the results (Takahashi et al., 2012; Degterev et al., 2013). It should be noted that RIP1 is involved also in apoptotic and survival signaling (Figure 2) so necrostatin-1 cannot be considered a specific inhibitor of necroptosis. Depletion of RIP3 or its substrate MLKL can also prevent necroptosis from taking place, favoring the apoptosis route (Chen et al., 2013; Wu et al., 2013; Remijsen et al., 2014). Finally, RIP3 may also play a role in the decision of the cell to follow an apoptotic or necroptotic route although the mechanism is unclear (Cho et al., 2009; Declercq et al., 2011; Tait et al., 2014).

### Necroptosis and the mitochondrion

Data implicating mitochondrial dysfunction in the execution of necroptosis is still very contradictory although the production of ROS and depletion of ATP support its involvement (Schulze-Osthoff et al., 1992; Leist et al., 1999; Zhang et al., 2009). The mitochondrial phosphatase PGAM5 has also been implicated in necroptosis. PGAM5 is a substrate of RIP3 and when activated, promotes the Drp1 translocation to the mitochondria whereupon it facilitates extensive mitochondrial division, ROS production and necroptosis (Wang et al., 2012; Zhang et al., 2013). However, recent evidence from PGAM5^−/−^ mice do not support its involvement in necroptosis (Moriwaki et al., 2016). Mitophagy (autophagic recycling of mitochondria) was recently implicated in the initiation of necroptosis. Inhibition of mitochondrial division or genetic ablation of PINK1, a protein kinase initiator of mitophagy, resulted in a decrease in necroptosis in an *in vivo* model of chronic obstructive pulmonary disease (Mizumura et al., 2014). However, this has been confounded by a study in cells in which mitochondrial number have been drastically reduced. TNFα induced necroptosis was performed in cells in which the mitophagy pathway was upregulated, ablating mitochondria in 80% cell population. No significant protection from cell death was observed (Tait et al., 2013). Clearly whether mitochondria are involved in the development of necroptotic cell death is still highly speculative and further work is required.

### Necroptosis and HI injury

The development of knockout mouse models of RIP3 and MLKL, has permitted the analysis of pathological necroptosis in a wide variety of injury models (Wu et al., 2013). A role for necroptosis-mediated cell death has been suggested in infection (Cho et al., 2009), inflammation (Duprez et al., 2011), pancreatitis (Wu et al., 2013), atherosclerosis (Lin et al., 2013) and ischemia-reperfusion injury (Linkermann et al., 2012; Oerlemans et al., 2012). Of relevance to this review, necroptotic cell death has been identified in both adult and immature brain, in response to ischemic injury. The original paper describing the discovery of necrostatin-1 found that after middle carotid artery occlusion generating a transient focal ischemia in rats, Necrostatin-1 treatment reduced infarct size whether administered pre- or post-injury (Degterev et al., 2008, 2013). This was recapitulated in a subsequent study where Necrostatin-1 was combined with anti-apoptotic drugs and showed protection in both *in vitro* oxygen/glucose deprivation experiments as well as in focal ischemia (Xu et al., 2010). Following intracerebral hemorrhage, both hematoma volume and neurovascular damage were also reduced by necrostatin-1 (King et al., 2014).

The role of necroptosis in immature brain injury has only recently been explored. Initial observations by Northington and colleagues suggesting that the morphological and molecular landscape of neonatal brain death is more of a “continuum,” ranging from apoptosis through necroptosis to necrosis (Northington et al., 2007). Using a neonatal mouse model of HI injury, injury progression was blocked, RIP1-RIP3 interaction prevented and NFκB and caspase-1 signaling inhibited after necrostatin-1 injection post-injury (Northington et al., 2011). Oxygen glucose deprivation (an *in vitro* mimic of HI) induced necroptotic cell death in primary hippocampal neurons, mediated by an upregulation of RIP3 expression and a transient decrease of caspase-8 (Vieira et al., 2014). This was mirrored *in vivo* after global cerebral ischemic insult in which RIP3 expression was similarly upregulated (Vieira et al., 2014). In acute neonatal injury, necrostatin-1 treatment reduced injury volume and improved behavioral outcomes in a model of traumatic brain injury (You et al., 2008). However, apoptotic signaling is also widespread following HI injury in neonatal mouse models (Hagberg et al., 2009) and necrostatin treatment not only inhibits necroptosis, but also alters cell death to a more apoptotic phenotype (Northington et al., 2011) supporting the idea that a continuum of cell death takes place depending on the injury environment. Necrostatin-1 decreased the accumulation of oxidants, prevented the decline in mitochondrial complex I activity and improved ATP levels 24 and 96 h after neonatal HI (Chavez-Valdez et al., 2012). A recent study of cell death after severe neonatal hypoxic-ischemic injury identified that although necroptosis was apparent at the core of the lesion, it was significantly higher in the peri-infarct region in severe injury compared with moderate injury (Askalan et al., 2015). A very recent study suggests that oxygen-glucose deprivation insult resulting oligodendrocyte cell death acts through a mechanism dependent on RIP3 upregulation. Oxygen-glucose deprivation induced the interaction between RIP3 and MLKL as well as RIP3 and CaMKII. Not only did interruption of these interactions mediate cell survival *in vitro*, but disturbing RIP3 interactions *in vivo* prevented myelination defects (Qu et al., 2017). Recently, endoplasmic reticulum (ER) stress has been suggested to be important in the necroptosis process. Neonatal HI induces shedding of dilated ER fragments in the cytosol and upregulation of ER stress markers and these alterations are reversed by Necrostatin-1 (Chavez-Valdez et al., 2016). Taken together, the emergence of these studies holds the tantalizing possibility of new neurotherapeutic targets to ameliorate HI-mediated neonatal brain injury.

### Autophagic cell death

In addition to apoptosis and necroptosis another, caspase-independent, mechanism of cell death has been proposed through overactivation of autophagy, a normally pro-survival mechanism of recycling cellular components. The criteria surrounding the definition of autophagic cell death is still debated (Kroemer et al., 2009; Galluzzi et al., 2012c, 2015) but autophagy is observed in a variety of physiological and pathological events, such as normal development, nutrient deprivation, neurodegeneration, immunity, and aging (Choi et al., 2013). Autosis is sometimes used synonymously with autophagic cell death. It is still unclear whether autosis is a subform of autophagic cell death that depends in Na^+^/K^+^-ATPase or if all forms of autophagic cell death relies on Na^+^/K^+^-ATPase (Liu and Levine, 2015) (see below).

### Autophagy

**Figure 3:**
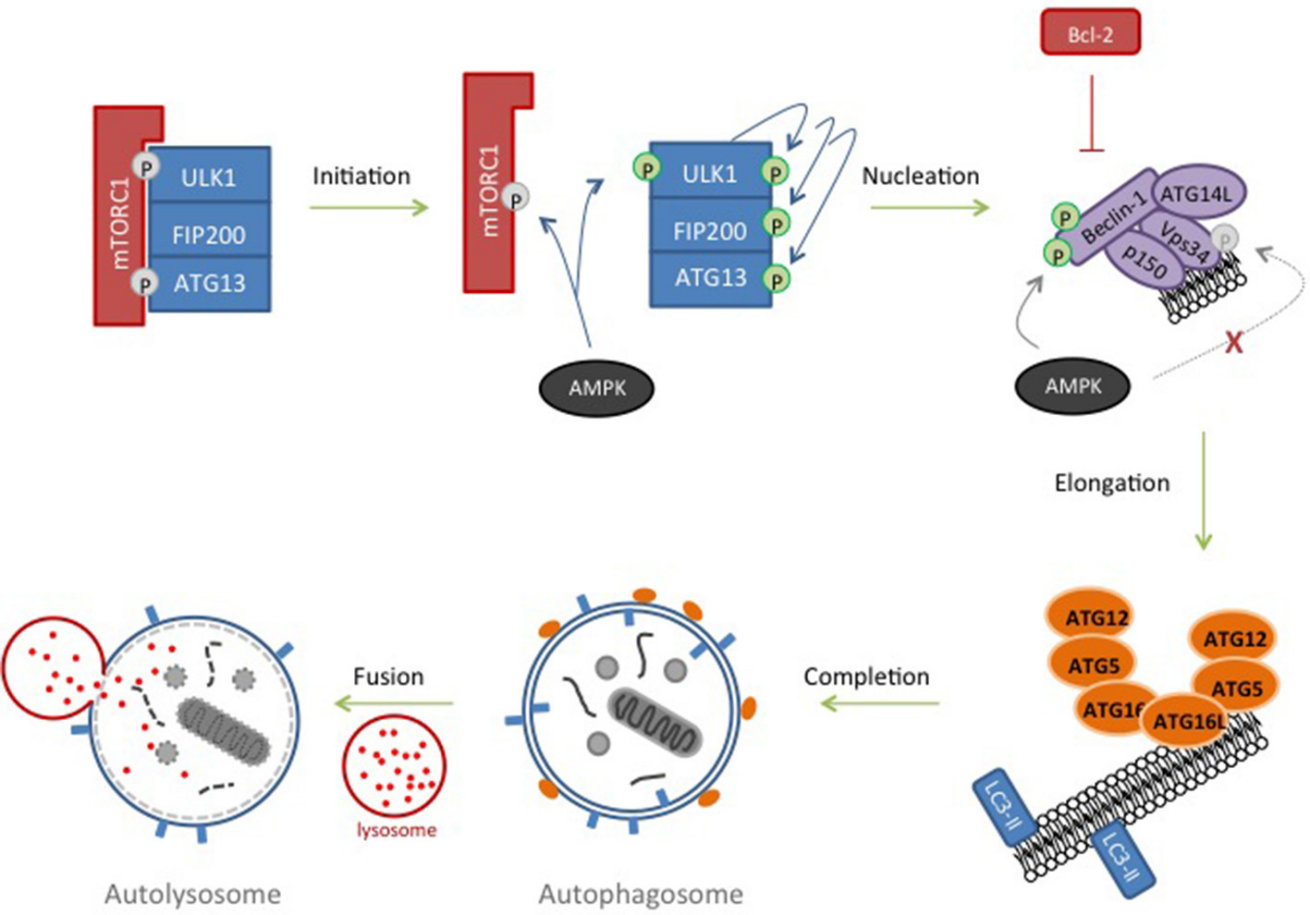
Autophagy. In response to nutrient deprivation, inhibition of ULK1-FIP200-ATG13 complex by mTORC1 is removed. ULK1 autophosphorylates and activates ATG13 and FIP200. AMPK, activated in response to starvation, contributes by phosphorylating and inhibiting components of the mTORC1 complex and phosphorylating and further activating ULK1. ULK1 subsequently phosphorylates Beclin-1 and Vps34 resulting in nucleation of the isolation membrane. Inhibitory phosphorylation of Vsp34 by AMPK is prevented by recruiting ATG14L to the complex and AMPK then phosphorylates and further activates Beclin-1. Lipids are recruited to the growing phagophore and two ubiquitin-like conjugation pathways are triggered resulting in an ATG12-ATG5-ATG16 complex at the autophagosome and LC3-II insertion into the membrane, where it recruits cargo. Lysosomes dock to the outer membrane of the autophagosome forming an autolysosome, and allowing hydrolases to degrade its contents.

Macroautophagy (subsequently referred to as autophagy) is a process in which proteins, protein complexes and even organelles are engulfed by an isolation membrane which extends to form an autophagosome. Once mature, the outer membrane of the autophagosome fuses with a lysosome to form an autolysosome, the cargo of which is degraded by lysosomal hydrolases (Marino et al., 2014; Figure 3). Autophagy is a highly conserved process (indeed, over 30 autophagy-related (ATG) proteins have been identified in yeast (Tsukada and Ohsumi, 1993; Suzuki and Ohsumi, 2007; Nakatogawa et al., 2009) and is initiated by a regulated interplay of phosphorylation and dephosphorylation. Autophagy is classically triggered in response to nutrient deprivation which promotes formation of the ULK1 pre-initiation complex comprising ULK1 (UNC-51-like kinase1)-FIP200 (FAK kinase interacting protein of 200 kD)-ATG13-ATG101 (Figure 3). In a nutrient-rich environment, this complex is normally inhibited by mammalian rapamycin sensitive mTOR complex (mTORC1) but during starvation, it is the mTORC1 which is inhibited (Chan, 2009; Hosokawa et al., 2009; Jung et al., 2009). Activation of ULK1 by phosphorylation results in activation of a phosphatidylinositol (PI)-3 kinase complex comprising Beclin1, Vps34, ATG14L, and p150 (Itakura et al., 2008), usually inactivated by anti-apoptotic BCL-2 family members (Pattingre et al., 2005). The nutrient-sensing protein AMP-activated protein kinase (AMPK) also plays a regulatory role throughout initiation and nucleation phases (Carling et al., 2012). Active AMPK phosphorylates and inactivates Raptor (Gwinn et al., 2008) as well as concomitantly activating ULK1 by phosphorylation (Egan D. et al., 2011; Egan D. F. et al., 2011). Subsequently, recruitment of ATG14L into the Beclin complex inhibits AMPK phosphorylation of Vps34 and promotes AMPK phosphorylation of Beclin-1 (Kim et al., 2013). Together with ULK1, the Beclin complex drives PI-3 phosphate formation and the nucleation of the isolation membrane by recruiting a number of ATG proteins to the emerging autophagosome (Figure 3). Transmembrane proteins, such as VMP1 and ATG9 interact with Beclin-1 and likely play a role in recruiting lipids to the autophagosome (Yamamoto et al., 2012; Molejon et al., 2013). At this point, two ubiquitin-like cascades are activated resulting in the conjugation of ATG5 to ATG12 at the outer membrane of the autophagosome and the conjugation of cytosolic microtubule-associated protein 1A/1B light chain 3 (LC3) with phosphatidylethanolamine. This converts it from LC3-I to LC3-II, whereupon it is inserted into the membranes of the rapidly closing autophagosome and acts to recruit cargo (Kabeya et al., 2000). This conversion from LC3-I to LC3-II and its subsequent relocalization is often used as experimental marker for autophagy as LC3-II remains membrane-associated until the end of the process. Finally SNARE proteins recruit and dock lysosomes to the outer membrane of the autophagosome resulting in formation of the autolysosome, influx of acid hydrolases and degradation of cellular contents (Longatti and Tooze, 2009).

### Autophagic cell death

Although the pro-survival function and benefits of autophagy are clear, extreme levels of autophagy have been proposed to trigger cell death. As is the case for the cell death field in general, the definition of autophagic cell death has recently been refined in order to move away from a classification simply based on morphology; accumulation of autophagosomes and autophagic vacuoles are also observed in response to apoptosis and necrosis (Galluzzi et al., 2012c). Autophagic cell death is now described as cell death suppressed by inhibition of the autophagy pathway as in some experimental systems, it has been hard to distinguish between autophagy causing cell death by triggering other cell death pathways (e.g., apoptosis, necrosis) and autophagy causing cell death itself (Levine and Yuan, 2005; Kroemer and Levine, 2008). Furthermore, a minimum of two components of the pathway need to be targeted as a number of proteins responsible for the execution of autophagy act in other, non-autophagic pathways (Galluzzi et al., 2015). Even with these stricter criteria, a number of examples of autophagic cell death can be observed in a variety of cell types and tissues. Embryonic fibroblasts from mice lacking the apoptosis regulators BAX and BAK underwent cell death after treatment with apoptosis-inducing agents (etoposide and staurosporine). However, this cell death was autophagic in nature, prevented by autophagy inhibitors and was characterized by autophagosome formation (Shimizu et al., 2004, 2010). Knockdown of ATG5 expression in HeLa cells results in resistance to cell death induced by interferon-γ treatment and conversely, over-expression results in autophagic cell death, even in the presence of a functioning apoptotic pathway (Pyo et al., 2005). Beclin-1 overexpression can be considered as facilitating autophagic cell death as knockdown of ATG5 prevents cell death (Pattingre et al., 2005). Ablation of beclin-1, ATG5 or ATG7 in transformed or cancer cell lines will prevent the induction of autophagic cell death in response to oxidative stress, such as H_2_O_2_ production (Chen et al., 2008). Furthermore, inhibition of caspase-8 or caspase-10 in certain cancer cell lines result in autophagic cell death although the mechanism of cell death is unclear; inhibition of catalases and a concomitant accumulation of ROS has been observed (Yu et al., 2004, 2006; Lamy et al., 2013). Very recently, another category of autophagic cell death has been proposed, termed “autosis.” Exposure of Hela cells to the cell-permeable Tat-Beclin peptide induced autophagic cell death with a distinct morphology—early nuclear convolutions, increased autolysosomes and later on, perinuclear swelling (Liu et al., 2013). Physiological stresses, such as starvation and hypoxia also induced a similar morphology although only in a small subset of the total cell population. Interestingly, this form of autophagic cell death is regulated by Na^+^, K^+^-ATPase as autosis can be inhibited by treatment with cardiac glycosides.

### Autophagic cell death and HI injury

Not only is autophagy activated due to neonatal nutrient deprivation (Kuma et al., 2004), but acute cellular events which occur during HI injury, such as calcium influx (Hoyer-Hansen et al., 2007) and ROS production (Chen et al., 2009) are also triggers for autophagy. It is therefore unsurprising that increases in autophagic flux and markers of autophagy are observed in rodent models of adult and neonatal HI (Zhu et al., 2005, 2006; Carloni et al., 2008; Balduini et al., 2009; Ginet et al., 2009). Whether this induction of autophagy is beneficial or deleterious to the animal is currently unclear (Levine and Yuan, 2005; Carloni et al., 2012, 2014) although recent evidence suggests the latter may be true. Characteristics of autophagic cell death have been observed in the absence of apoptotic markers in various models of neonatal HI (Puyal and Clarke, 2009; Puyal et al., 2009) and studies of hippocampal slices exposed to OGD showed that pharmacological inhibition of autophagy ablated neuronal cell death (Lu et al., 2015). In support of this, a recent study into mice lacking *Atg7* showed evidence of reduced neonatal brain injury after HI (Xie et al., 2016), interestingly there was an obvious inhibition of both caspase-dependent and -independent cell death in multiple brain regions. *In vivo*, pharmacological inhibition of autophagy prior to induction of HI prevented the increase in LC3-II as well as reducing memory impairment in behavioral tests (Xu et al., 2016). Finally, melatonin treatment administered just prior to and subsequently after HI in rat pups conveyed neuroprotection through mechanisms targeting both apoptotic and autophagic cell death (Hu et al., 2017).

These recent findings are in line with previous studies suggesting brain region- and gender-specific differences in induction of autophagic cell death after neonatal brain injury (Zhu et al., 2005, 2006; Koike et al., 2008; Weis et al., 2014). Furthermore, it was recently shown that autosis, dependent on Na^+^, K^+^-ATPase, was detected regionally in the hippocampus after neonatal HI (Liu et al., 2013).

## Conclusion

Previously, it was believed that cells died either through accidental necrosis or regulated (programmed) apoptotic cell death. Today it is becoming generally accepted that there are several forms of regulated cell death (e.g., apoptosis, autophagic cell death/autosis, necroptosis, parthanatos), defined by biochemical hallmarks rather than (only) morphological features. Indeed, recent experimental studies suggest that accidental as well as most of the above mentioned types of regulated cell death pathways are important in the context of immature brain injury depending on the intensity and type of insult, cell type, brain region and developmental age. Furthermore, if one mode of cell death is inhibited, another route may step in provided that the upstream triggering forces are sufficiently strong. The provision of alternative routes through which the cell can succumb to death has to be taken into consideration in the search for novel neuroprotective strategies.

## Author contributions

HH and CT conceptualized and designed the review and drafted the initial manuscript. BL, CM, SN, and MJ all assisted in the careful assessment of each of the papers cited in the review. All authors took part in the critical interpretation of the scientific data, phrasing of text and designing the figures and table. HH obtained the major part of the funding to support the work and all authors approved the final manuscript as submitted and agree to be accountable for all aspects of the work.

### Conflict of interest statement

The authors declare that the research was conducted in the absence of any commercial or financial relationships that could be construed as a potential conflict of interest.

## Abbreviations

AIF: apoptosis-inducing factor
Akt: also called protein kinase B
AMPA: α-amino-3-hydroxy-5-methyl-4-isoxazolepropionic acid
AMPK: AMP-activated protein kinase
APAF-1: apoptotic peptidase-activating factor-1
ASK1: apoptosis signal-regulating kinase
ATG: autophagy related
ATP: adenosine triphosphate
BAD: BCL2 associated death promotor
BAK1: Bcl-2-antagonist/killer 1
BAX: Bcl-2-associated X protein
BCL2: B-cell lymphoma 2
BCL- X_L_: BCL2 like 1
BID: BH3- interacting domain death agonist
BIM: BCL2-like 11
CAMKII: calcium/calmodulin dependent protein kinase 2
cIAP: cellular inhibitor of apoptosis proteins
CNS: central nervous system
CYLD: Cylindromatosis
Cyt c: cytochrome c
CDK5: Cyclin-dependent kinase 5
DAMPs: damage-associated molecular patterns
DISC: death-inducing signaling complex
DR: death receptor
DRP1: dynamin-related protein 1
Endo G: endonuclease G
ER: endoplasmic reticulum
ETC: electron transport chain
FADD: Fas-associated death domain
FasL: Fas ligand
Fn14: fibroblast growth factor inducible 14
FIP200: FAK kinase interacting protein of 200kD
FOXO3a: Forkhead box O3a
GSK3β: glycogen synthase kinase-3β
HI: hypoxia-ischemia
HIF-1a: hypoxia inducible factor1 alpha
IAP: inhibitors of apoptosis
IFN: interferon
IL: interleukin
JNK: c-Jun N-terminal kinase
LC3: microtubule-associated protein light chain 3
MAPK: mitogen activated protein kinase
MCL1: myeloid cell leukemia 1
MDM2: Mouse double minute 2
MLKL: mixed lineage kinase domain-like
MOMP: mitochondrial outer membrane permeabilization
MPT: mitochondrial permeability transition
mTORC: mammalian rapamycin sensitive mTOR complex
NFκB: nuclear factor kappa B
NMDA: N-methyl D aspartate
NOXA: Phorbol-12-myristate-13-acetate-induced protein 1
OPA1: optic atrophy 1
PARP-1: poly (ADP-ribose) polymerase 1
PGAM5: phoshoglycerate mutase family member 5
PI: phosphatidylinositol
PIDD: p53-induced death domain-containing protein
PINK1: PTEN-induced putative kinase 1
PTEN: phosphatase and tensin homolog deleted on chromosome 10
PUMA: pro-apoptotic members BCL2 binding component 3
Q-VD-OPh: quinoline-Val-Asp(Ome)-CH2-O-phenoxy
RIP: receptor-interacting kinase
RAIDD: RIP associated ICH-1/CED3 homologous protein with a death domain
RHIM: RIP homotypic interaction motif
ROS: reactive oxygen species
SMAC: second mitochondria-derived activator of caspases
SNARE: soluble N-ethylmaleimide-sensitive fusion (NSF) attachment protein receptors
tBID: truncated BID
TLR: Toll-like receptor
TNF-α: tumor necrosis factor alpha
TNF-R: TNF receptor
TRADD: Tumor necrosis factor receptor type 1-associated DEATH domain protein
TRAIL: TNF-related apoptosis-inducing ligand
TRIF: TIR-domain-containing adapter-inducing interferon-β
TWEAK: tumor necrosis factor-like weak inducer of apoptosis
ULK1: UNC-51-like kinase1
VDAC: voltage-dependent anion channels
VMP1: vacuole membrane *protein* 1
VPS: vacuolar protein sorting.
